# Multimodal HSI Combined with Multiblock Data Fusion: A New Tool for the Study of Time-Dependent Alteration Processes in Dyed Textiles

**DOI:** 10.1021/acs.analchem.4c04236

**Published:** 2024-12-06

**Authors:** Zelan Li, Alessia Candeo, Emilio Catelli, Marta Ghirardello, Paolo Oliveri, Cristian Manzoni, Silvia Prati, Daniela Comelli, Giorgia Sciutto

**Affiliations:** 1 Department of Chemistry “Giacomo Ciamician”, University of Bologna, Via Guaccimanni 42, 48121 Ravenna, Italy; 2 Department of Physics, Politecnico di Milano, Piazza Leonardo da Vinci, 20133 Milan, Italy; 3 Department of Pharmacy, University of Genoa, Viale Cembrano 4, I-16148 Genoa, Italy; 4 IFN-CNR, Piazza Leonardo da Vinci 32, 20133 Milano, Italy

## Abstract

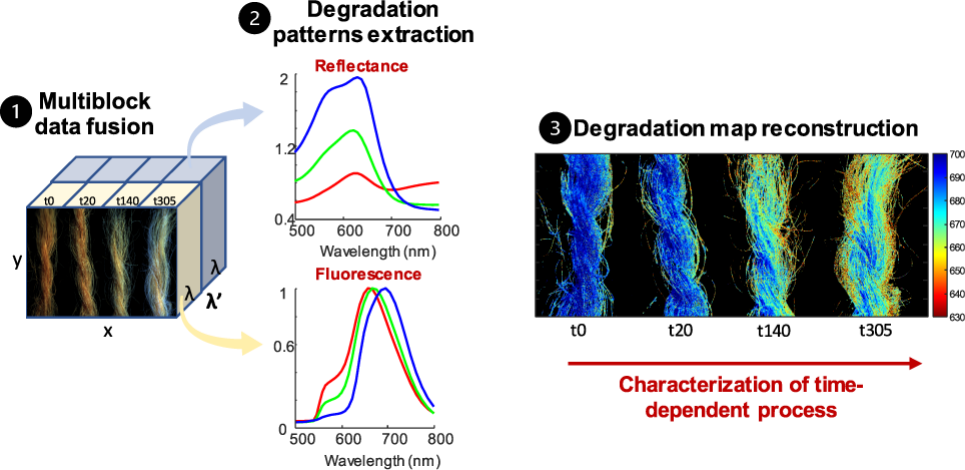

The present study describes an innovative approach for the study of time-dependent alteration processes. It combines an advanced hyperspectral imaging (HSI) system, to collect visible reflectance and fluorescence spectral data sets sequentially, with a tailored multiblock data processing method. This enables the modeling of chemical degradation maps and the early, spatially resolved detection of dye alteration in textiles. A chemometric method based on data fusion and principal component analysis was employed to identify spectral features of dye degradation, combining and enhancing information from reflectance and fluorescence HSI data. The most significant spectral profiles extracted were used to develop an asymmetric Gaussian-based pixel-by-pixel fitting model applied to the HSI fluorescence data set, enabling the reconstruction of degradation maps for rapid and intuitive visualization. In particular, changes in intensities and horizontal shift of dye emission peaks were pixel-by-pixel evaluated and fitted for the reconstruction of the degradation maps. Artificially aged wool samples tinted with indigo carmine (IC) dye served as a case study. IC is extensively used in textiles, and it is notable for its light sensitive. The results show that this approach effectively identifies spatial variations and chemical changes in dyed wool fibers, offering potential for sustainable conservation of historical textiles and other types of time-dependent processes. Thus, by amplifying variation in spectral profiles induced over time by aging, even minimal changes at early stages can be easily detected and localized, offering powerful tools for future studies on food and drug shelf life and stability, as well as forensic trace analysis.

## 1 Introduction

Natural dyes have played a crucial role in the textile industry since antiquity, contributing significantly to the aesthetic and historical value of cultural heritage objects.^1^ Dyes, however, are susceptible to degradation, which poses considerable challenges in conservation planning and maintaining of the historical integrity of textile-based artifacts. The development of enhanced analytical methods capable of monitoring the conservation state for early detection of degradation is urgently needed to facilitate effective preservation and appropriate conservation strategies. The degradation process of dyes, induced by factors such as light, heat, humidity, and biological agents, profoundly affects the appearance, integrity, and longevity of dyed materials, resulting in color fading, alteration, or even complete loss.^2−4^ Therefore, in the field of textile conservation, accurate analysis of dyes, their aging processes, and chemical degradation pathways is significantly important. Several studies have reported the chemical analysis of dyes in textiles using analytical techniques based on sample extraction with a focus on the chemical characterization of dyes and their degradation processes. In recent decades, significant progress has been made in reducing the sample size required for analysis, with current methods needing only a few micrograms.^5^

High-performance liquid chromatography is typically the preferred technique, and an extensive body of literature exists on its utilization for dye analysis.^3,6−10^

High-sensitivity spectroscopic techniques have also been proposed. Surface enhanced Raman spectroscopy (SERS), which exploits the amplification of the Raman signal when organic molecules are adsorbed onto nanometric rough metallic substrates,^11^ has gained prominence for the detection of natural^12−14^ and synthetic^15,16^ dyes in historical textiles. *In situ* SERS, combined with gels for dye extraction, has been proposed to further limit the invasiveness of the method by minimizing the amount of dyes extracted from textiles.^17,18^ Metal underlayer attenuated total reflection (MU-ATR-FT-IR) has been reported as a successful method to enhance the sensitivity of FT-IR spectroscopy in dye analysis.^19,20^ Finally, the combination of multiple approaches, as thin layer chromatography, MU-ATR-FT-IR, and SERS spectroscopies, has been explored for the identification of trace amounts of dyes in mixtures.^21^

Fluorescence spectroscopy, based on the unique fluorescence characteristics of organic dyes, provides insights into the dye molecular structure and its interactions with surrounding molecules.^22−24^ When used to study dyed wool fabrics and yarns, it has proved to be able to provide a specific spectral fingerprint of dyes by employing multiple excitation wavelengths through the collection of the information-rich excitation–emission matrix data set.^25,26^ UV–VIS reflectance spectroscopy is another noninvasive technique useful to get information on the molecular composition of a sample, and when used for dye analysis, the method not only identifies the inherent color of dyes but also detects subtle color changes linked to degradation.^27,28^ Importantly, these techniques are both noninvasive and can be easily implemented as imaging methods thanks to the advent of hyperspectral imaging (HSI), which open up new possibilities for the chemical mapping of dyes in textiles.^29−33^ Further, considering its easy implementation without the need for any contact with the artifact under analysis, HSI offers a tool to monitor degradation over time and identify initial signs of fading and discoloration.

Within this scenario, the present research proposes an innovative multimodal integrated HSI approach for the characterization of the time-dependent process. The approach combines an advanced HSI system, which integrates visible reflectance and fluorescence spectroscopy, with a tailored multiblock data processing method for the reconstruction of degradation maps. The latter are achieved through the proper physical modeling of fluorescence spectral changes induced by degradation in artificially aged wool samples tinted with indigo carmine dye. The approach negates the need of sample collection and combines the study of HSI data sets of light diffusely reflected and emitted by samples in the VIS–NIR spectral range. This enables the sequential collection of spatially correlated multimodal data sets and supports data fusion without the need of image registration procedures.

**Figure 1:**
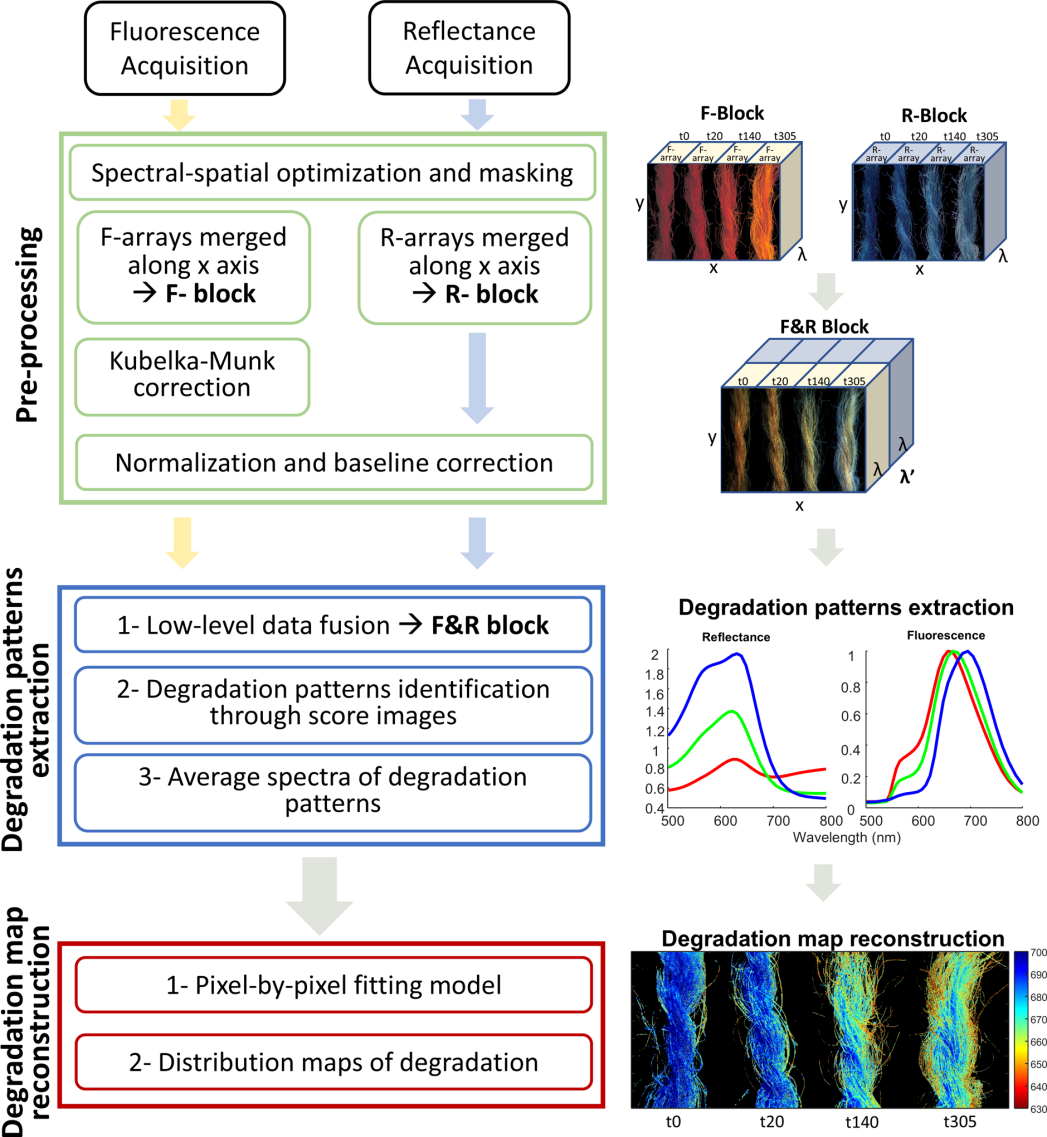
Workflow of multiblock data analysis following multimodal HSI data sets acquisition.

To enhance the extraction and integration of the information embedded in reflectance and fluorescence HSI data, a chemometric-based multiblock data processing approach, based on data fusion and principal components analysis (PCA), was first adopted to extract spectral features associated with dye degradation at different stages. Results of chemometric analysis were then used as reference spectral profile for the setup of a fitting Gaussian-based mathematical model, subsequently applied on the HSI fluorescence data set on a pixel-by-pixel basis. The final outcome are chemical maps related to dye degradation, which, with rapid and intuitive visualization, provide the immediate perception of dye degradation at early stages and a clear view of how degradation impacts on the different parts of wool threads (Figure 1).

The application of the proposed approach focused on a specific dye, indigo carmine (IC), also known as Saxon Blue (the molecular structure is shown in Supporting Information Figure S1). The dye was originally derived from the plant-based indigo during in the 18th century and was considered to be the first semisynthetic dye.^3,34^ The widespread usage of IC in textiles and in various fields ranging from the food industry to biomedical research^35−38^ makes it a dye of high interest to researchers. Besides, IC is notably more sensitive to light than its precursor, indigo, and its increasing sensitivity highlights the necessity for in-depth studies of its degradation behavior.

## 2 Materials and Methods

### 2.1 Sample Preparation

Dyed wool fiber samples (Bluefaced Leicester, brand: Rowan, Purelife) were prepared using the recipe published by Adolf Lehne in 1893, implemented at the OCW/Rijksdienst voor het Cultureel Erfgoed (The Netherlands). The wool fibers were cleansed with 10% (weight relative to wool) Marseille soap at a temperature of 40 °C before the dyeing process commenced.

A portion of the wool fibers was left undyed to allow characterization of the wool optical properties (with reference to the wool emission spectrum), while the remaining portion was dyed using the procedure described below.

For dyeing with IC, purchased from Sigma-Aldrich, 750 mL of demineralized water was heated to and maintained at 70 °C throughout the dyeing process. To this heated solution, 5 g of sodium sulfate (Na_2_SO_4_) was added and dissolved. Subsequently, 150 mg of IC was measured and introduced into the solution. A 10 g bundle of wool was immersed in this dyeing solution. Thereafter, 200 μL of concentrated sulfuric acid was added to the beaker. The dye bath was then placed in an oven set to 70 °C for 30 min. To ensure a uniform color, the dyeing solution was agitated every 5 min. Following the dyeing process, the bundle was allowed to cool in the dye bath and was later rinsed with demineralized water.

Artificial aging was carried out by Dr. Witold Nowik from the Département Recherche, Centre de Recherche et de Restauration des Musées de France (C2RMF) as part of the IPERION project (Grant Agreement ID: 654028). A Xenotest 150 S (Heraeus) chamber, equipped with a 1300 W air-cooled xenon arc lamp, was used for the aging process. To simulate the effect of solar irradiation through glass, lamp emission was limited in the spectral range 310–830 nm with the aid of a UV cutoff filter at 310 nm and an IR blocking filter, giving rise to a total irradiance of 1154 W/m^2^. The chamber temperature was kept at 40 °C, while relative humidity was left uncontrolled. Samples were mounted on rotating biface holders with an aluminum foil-covered reflective support. The exposure mode was alternated (one full rotation exposed, followed by a 180° holder rotation). Samples were collected before aging (*t* = 0 h) and after 20, 140, and 305 h of artificial aging.^39^ The same aging procedure was applied to undyed wool samples to assess the possible degradation of the wool itself.

### 2.2 Instrument Setup and Data Acquisition

The HSI camera is a Fourier transform-based system employing the TWINS interferometer coupled with a monochrome camera, as described in detail in past publications.^40,41^ The TWINS (translating-wedge-based identical pulses encoding system) interferometer is based on an ultrastable, compact, and common-path scheme. When combined with an imaging system, it enables performing HSI with high robustness, stability, versatility, and compactness. The system has a spectral resolution of up to 4 nm @ λ = 600 nm and keeps optical alignment even in the presence of mechanical perturbations, without any active control. Thanks to these features, the system has been used for large field-of-view imaging,^40^ even for *in situ* experiments,^41^ for microimaging in different modalities,^42,43^ and very recently, for macroimaging.^32^ The latter configuration is shown in Supporting Information Figure S2 and employs a quasi-4f optical system in the collection path, made of a reversed photography Nikon objective (Nikon Nikkor, effective focal lens = 50 mm, F#1.2) and a 25 mm camera lens (Thorlabs MVL25M23, effective focal lens = 25 mm, F#1.4) with the TWINS interferometer placed between the two objectives. In this implementation, the FOV is 1.2 mm wide, the working distance is 46.5 mm, and the setup displays a spatial resolution of 30 μm.

Each IC wool sample was sequentially analyzed in terms of its reflectance and fluorescence spectral properties. The former study was achieved by illuminating the wool sample with a halogen lamp (Leica CLS 100× Microscope Cold Light Source, irradiance of 3 W m^–2^ corresponding to 630 lx). Illumination was delivered through two optical fibers placed at the sides of the sample, at an angle of 45° with respect to the collection. To reconstruct diffuse reflectance spectral data, spectral calibration was achieved with an absolute-standard-white reference (Labsphere Spectralon, >95% total reflectance in the range 250–2500 nm). In the case of fluorescence analysis, the excitation light was the second harmonic of a Nd:YAG laser (λ = 532 nm, average power = 1 mW, FTSS 355-50 CryLas GmbH), delivered to the sample through a multimode silica fiber (core diameter = 600 μm) coupled to proper optical system^32^ to achieve a flat-top circular illumination with a diameter of about 2 cm. The emission from the sample was filtered along the detection path with a long pass filter at 550 nm (Thorlabs, FELH550).

### 2.3 Multiblock Data Analysis

After HSI data acquisition, two HSI data arrays were obtained for each individual sample: a fluorescence data array (F-array) and a reflectance data array (R-array), with the same spatial (1024 × 1100 pixels) and spectral (400–1000 nm, 75 variables) dimensions.

Data analysis protocol involved (i) different preprocessing operations, (ii) spatial segmentation into clusters associated with different levels of IC degradation, and (iii) the modeling of fluorescence spectral data to map IC degradation on fiber wool samples on a pixel-by-pixel basis.

All multiblock data analysis procedures were conducted using MATLAB (R2022b, MathWorks, Inc.).

#### 2.3.1 Multiblock Data Preprocessing

Data preprocessing operations can be divided into two main steps.

The first step involved removing noninformative data from each individual data array by spectrally cropping all data set in the spectral ranges of interest (500–800 nm for both the R-array and the F-array). A spatial mask was created by identifying noninformative areas in the F-array and R-array. For the purpose, k-means clustering was employed to the F-array to identify nonfluorescent areas. Instead, in the R-array, thresholding was applied to identify and neglect pixels with reflectance values higher than one, due to points in samples where specular reflected light was detected. All these pixels were considered as nonsignificant and were disregarded in the following procedures by setting them to NaN (not-a-number).

In a second step, data arrays related to different aging times were spatially stitched. This resulted in a single fluorescence (F-block) and reflectance (R-block) block containing all four samples. By using the absorption information contained in the R-block, the F-block data set was corrected for self-absorption phenomena with a well-known model based on the Kubelka–Munk theory of light diffusing in opaque samples. The Kubelka–Munk correction is used to adjust the fluorescence emission spectra for self-absorption, utilizing the diffuse reflectance spectrum to reconstruct the true fluorescence. This correction procedure is widely recognized as an effective method to estimate the true emission spectrum of a fluorescent material in paintings and artworks when self-absorption or absorption by surrounding chromophores occurs.^44^ On its turn, values of the R-block were converted from reflectance to pseudoabsorbance values. Row preprocessing was then performed on both blocks to correct for baseline shifts and drifts.^45^ Specifically, for the F-block intensity data scaling between 0 and 1 was followed by standard normal variate (SNV) transform and first derivative with the Savitzky–Golay algorithm (polynomial order 3, datapoint window size 7). For the R-block, a row-profile correction was performed to remove global intensity (multiplicative) effects.^46^ As the final step, a low-level data fusion method was chosen to merge F- and R-block by concatenating them along the spectral dimension after separate block autoscaling. The final data set is quoted in the following as F&R-block.

#### 2.3.2 Multiblock Data Segmentation

Image segmentation was then performed to classify pixels into clusters related to different levels of IC degradation. For the purpose, the F&R block was submitted to multivariate analysis. PCA, known for its efficiency in data reduction, was implemented on the whole F&R block, and the first score map (PC1) was considered for data segmentation by clustering it into three spatial clusters on the basis of the frequency distribution of PC1 score values. A false-color RGB image was then created to provide a quick visualization of the spatial segmentation process. As the segmentation procedure was applied to the whole the F&R block, its outcome was able to summarize the information gathered from both fluorescence and reflectance. In addition, average reflectance and fluorescence spectra were extracted from each segmented cluster to evaluate their spectral differences and use them as references for the fitting models.

#### 2.3.3 Fluorescence Block Data Fitting

Modeling of fluorescence spectral data, already corrected for self-absorption phenomena, was performed to assess chemical changes indicative of IC degradation. For the purpose the emission of dyed wool strands, *F*(λ) was modeled as the superposition of the emission of IC, *f*_IC_(λ), and the one of wool, *f*_W_(λ).
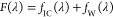


Each one of the two emissions was represented by a skew normal distribution to consider spectral asymmetries in the emission spectral profiles, as stated by the following formulas:
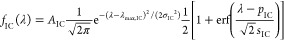

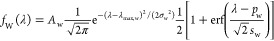


In these formulas *A*, λ_max_, and σ are the amplitude, the mean wavelength, and the standard deviation of the normal distribution, while *p* and *s* are the location and shape parameters of the error function *erf* accounting for the degree of asymmetry of the normal distribution. The subscripts IC and w refer to the emission of the dye and of the wool, respectively.

On its turn, the emission of the undyed wool sample was modeled as a single skew normal distribution, *f*_W_(λ).

The fitting procedure was first applied to the average emission spectrum of undyed wool to assess the parameters modeling its emission (specifically λ_max_, σ_w_, *p*_w_ and *s*_w_).

Once these parameters were identified, it was possible to fit the emission of dyed wool strands at different degradation stages by fixing λ_max,w_, σ_w_, *p*_w_, and *s*_w_ at the values previously found and letting all other parameters vary (specifically, the wool emission amplitude, *A*_w_, and all the parameters describing IC emission, *A*_IC_, λ_max,IC_, σ_IC_, *p*_IC_, and *s*_IC_). This procedure was first applied to the average emission spectrum of each cluster identified by the PCA-based segmentation procedure. Results of this procedure were then used to set proper initial values and boundary conditions of the fitting parameters for the subsequent pixel-by-pixel fitting procedure applied on the whole F-block.

Results of this latter fitting procedure were visualized in the forms of two maps related with the degree of degradation of dyed wool fibers, specifically the ratio among the emission amplitude of wool and IC, and the peak wavelength of IC.

## 3 Results and Discussion

### 3.1 Extraction of Degradation Patterns

**Figure 2:**
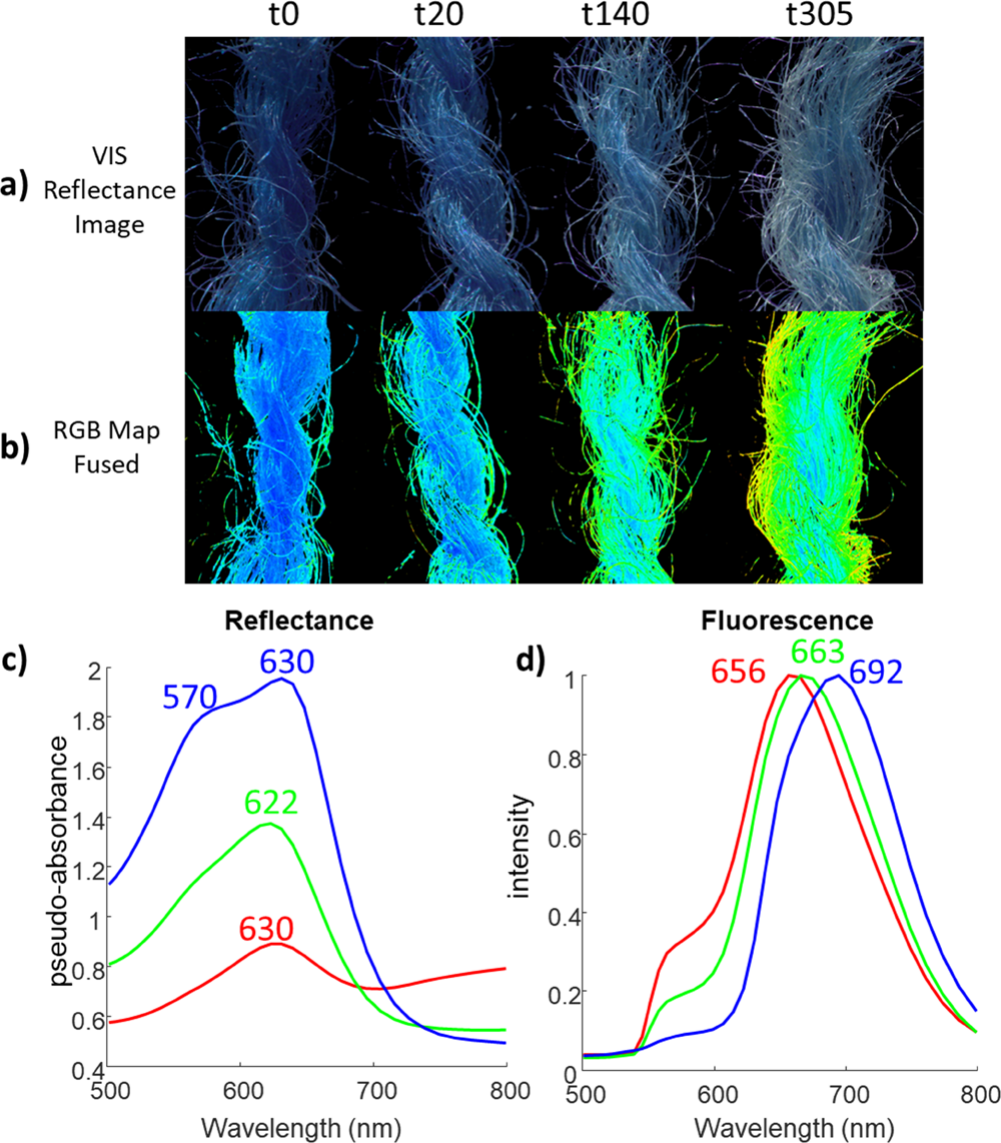
(a) Image of the four wool skeins reconstructed from HSI diffuse reflectance data in the spectral range 420–720 nm; (b) image segmentation achieved on the basis of PCA of the F&R multiblock and displayed as a false-color RGB map, where the blue, green, and red color are associated respectively with low, medium, and high PC1 score values; (c, d) average pseudoabsorbance and fluorescence spectra of the blue, green, and red cluster.

Following data acquisition and data preprocessing, PCA was undertaken on the whole F&R block created by low-level data fusion method of the four wool skeins exposed to different aging times (t0, t20, t140, and t305). PC1 expressed 41% of the total variance of the data set, and considering its high informative content, it was used alone to perform data clustering. The selection is further supported by the loading profile (Figure S3 in Supporting Information), which explains the influence of variables corresponding to the maximum absorption band of IC dye. Threshold values were selected by considering the local minima and the inflection points in the histogram of PC1 score values (Figure S4 in Supporting Information) and then coded with an RGB chromatic scale, obtaining a false-color RGB map: high scores, corresponding to values exceeding 1.8, were coded in red; medium scores, ranging from −0.2 to 1.4, were coded in green; low scores, ranging from −0.6 to −2, were coded in blue. The false-color RGB map (Figure 2b) revealed the presence of volumetric inhomogeneities in the wool yarn induced by aging, with well-preserved areas associated with low PC1 score values (blue color), highly degraded areas associated with high PC1 score values (red color), and areas of intermediate degradation state associated with medium PC1 score values (green color).

High color fading effects were observed especially for samples submitted to long exposure time, while the unaged sample t0 presented a quite homogeneous blue color in the false-color RGB map, signifying well-preserved IC.

Interestingly, undegraded IC was noted not exclusively in the unaged sample but was also discernible in the inner parts of aged samples. This observation suggested that the core of the wool skein was less degraded due to a lower exposure to UV light.

The average pseudoabsorbance spectra of the three clusters provided preliminary information on the progressive fading of IC over time (Figure 2c). The literatures report the maximum absorption peak of IC around 610 nm, which decreases significantly in intensity as the dye undergoes photodegradation.^47−49^ The extracted spectra showed a net decrease of the absorption band at 630 nm moving from undegraded to highly degraded fibers, coupled with increased absorption values toward the red-to-NIR spectral region in the high-degraded cluster, indicating the fading of the dye color.^50,51^ Worth mentioning, two bands were observed at 570 and 630 nm in the average spectrum of blue cluster (well-preserved fibers). The presence of these two bands might be attributed to vibrational progression and electronic transition (specifically a π–π* transition) of IC, representatively.^52^

The average fluorescence spectra of the three clusters provided further information on the degree of IC degradation in wool fibers. In the blue cluster the spectrum (Figure 2d) exhibited a distinct emission band peaked around 690 nm, which, in the green and red clusters, was shifted toward shorter wavelengths. This spectral shift is consistent with chemical changes associated with IC degradation and is indicative of alterations in the IC molecular structure.^51,53,54^ Similar shifts have been reported in previous studies focused on the evaluation of IC dye behavior in solution, strongly related to chemical modifications driven by solvent interactions, highlighting structural changes in the dye.

In addition, average fluorescence spectra of green and red clusters presented a shoulder between 550 and 600 nm ascribable to wool, whose optical emission becomes relevant following loss of dye color in the wool fiber. Undyed wool samples revealed subtle changes in their emission spectrum following aging, specifically a narrowing of the emission spectrum of a few nanometers (Figure S5 Supporting Information). These minor modifications have no effect on the spectral changes discussed in the dyed wool fibers. Therefore, the alterations highlighted in the spectrum of the dyed wool fibers should be attributed to IC degradation.

### 3.2 Modeling of Degradation Patterns

The averaged fluorescence spectra obtained from multivariate multiblock analysis allowed the definition of a proper spectral model for the emission of dyed wool fibers as the superposition of the emission of IC and of the wool, both modeled as asymmetric normal distributions.

To properly characterize the spectral emission of the wool, whose spectral features are partially hindered by IC emission in dyed wool fibers, the true fluorescence spectrum (corrected for self-absorption phenomena) was retrieved as the average spectrum of the undyed wool sample. Results of its nonlinear fitting as a single skew normal distribution is provided in Supporting Information (Figure S6 and Table S1), stating a normal distribution centered at about 580 nm with a spectral width of 95 nm and a skew modeled by an error function located at 550 nm with a shape parameter of about 10 nm. The asymmetry in the wool emission spectrum is mostly due to the filtering effect of the high-pass filter placed in the collection path of the HSI camera, which prevents the collection of the entire spectral emission of the wool.

Once the parameters describing the wool spectral emission were identified, the nonlinear fitting procedure was applied to the three average fluorescence spectra identified through PCA-based clustering, by modeling the emission as the superposition of two skew normal distributions (as detailed in section 2.3.3). Results of nonlinear fitting are reported in Figure S7 and Table S2 in Supporting Information. The emission of undegraded IC, well represented by the blue cluster, displayed an emission peak at 692 nm with a spectral width, described in terms of standard deviation of the normal distribution, of 70 nm. The emission is spectrally asymmetric, with the location and shape parameter found at around 630 and 20 nm, respectively. Spectral changes induced by IC degradation were quantitatively assessed following nonlinear fitting of the green and red clusters: (i) the IC emission peak was hypsochromically shifted from 692 nm to around 660 and 635 nm in the green and red cluster; (ii) the relative amplitude of wool emission with respect to the one of the IC showed a net increase from around 0.05 (blue cluster) to 0.2 (green cluster) and 0.3 (red cluster). The latter phenomenon is indicative of IC fading and results from the simultaneous decrease in IC emission intensity and increase in wool emission, which is absorbed to a lesser extent by IC molecules.

**Figure 3:**
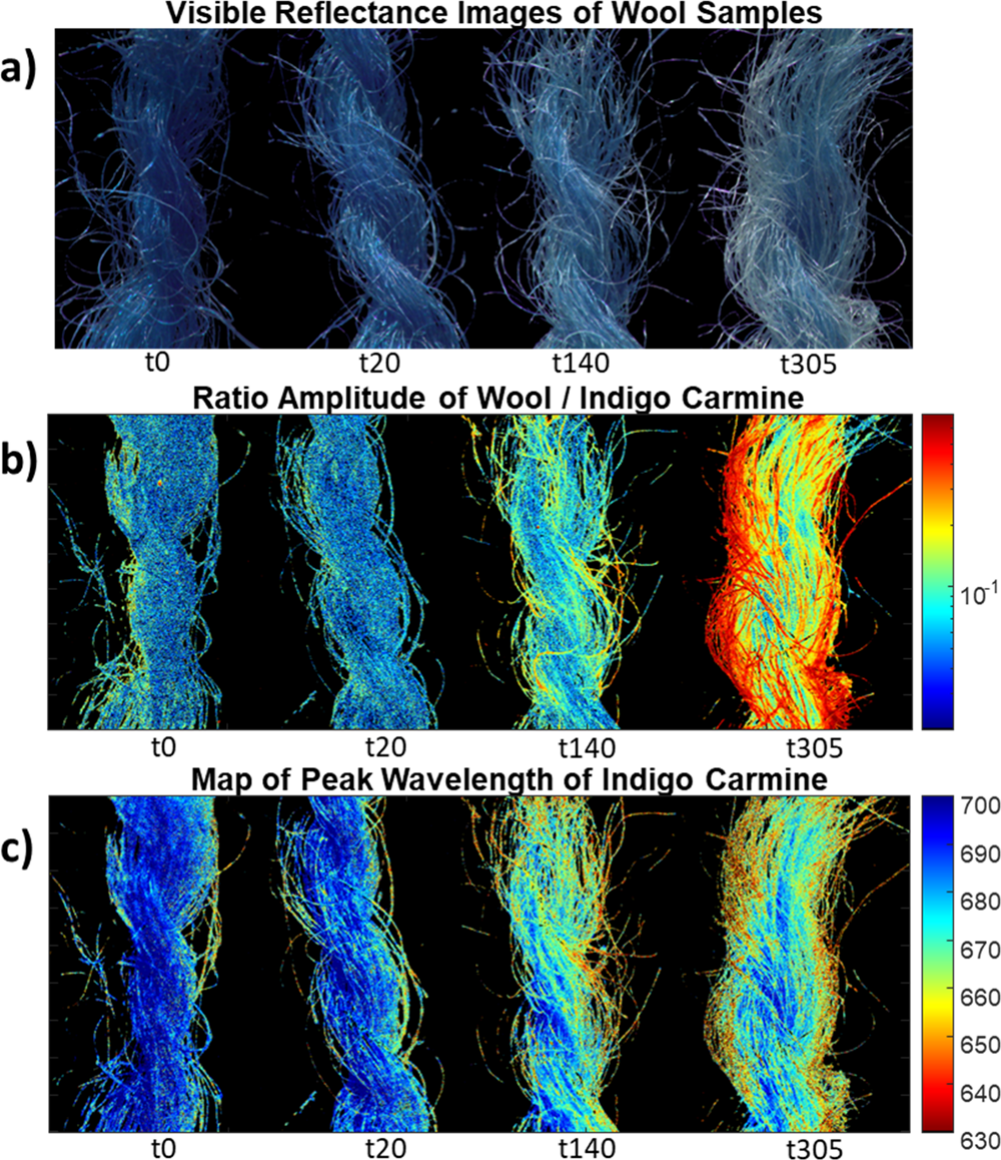
Degradation maps were generated by the pixel-by-pixel fitting model: (a) image of the four wool skeins reconstructed from HSI diffuse reflectance data in the spectral range 420–720 nm; (b) map of the ratio between the emission amplitude of wool and the one of IC; (c) map of peak wavelength of IC emission generated by the pixel-by-pixel fitting model.

The analysis of the average behavior of the emission of dyed wool fibers at different degradation stages allowed us to define the proper boundary and initial conditions (Table S3 in Supporting Information) for the nonlinear fitting of the whole F-block on a pixel-by-pixel basis. This final fitting procedure produced two degradation maps (Figure 3b,c) informing on the extent of IC degradation across the samples: the ratio of the amplitude of wool emission to that of IC, which quantifies their contributions to the overall optical emission and mainly describes the effect of dye fading, and the IC emission peak, whose spectral shift is related to the chemical modification of the IC conjugated system induced by degradation. Considering the ratio emission amplitude map (Figure 3b), early fading of the outer fibers was observed in the samples at the initial stages of degradation (t0 and t20). This effect became much more pronounced and spatially extended in the samples at t140 and t305. In terms of the spectral shift of IC emission, as the temporal extent of artificial aging progresses and the dye degrades, the hypsochromic shift of peak wavelength was observed in the outer fibers (Figure 3c). While for samples at t0 and t20 the emission peak was rather uniform throughout the two samples and close to a value of 690 nm, at t140 and t305 a net change of the emission peak was apparent in most of the fibers, apart for the ones mostly protected by the interweaving of wool threads.

As shown by the transition from t0 to t305, a detailed examination of these two different degradation maps further supports the identified degradation trends, which also reveals the presence of an early degradation state of the reference (unaged) sample. Interestingly, the two degradation maps did not display the exactly same spatial pattern on the analyzed samples, indicating that the changes in dye color and intensity relative emission are not strictly attributable with chemical modifications of the IC conjugated system.

## 4 Conclusion

The current research proposes new effective tools for the characterization of time-dependent processes, exploiting spectral features and providing spatially resolved information. The new instrumental setup was demonstrated to be suitable to obtain chemical images from different spectroscopies with high spatial coherence for multiblock data fusion and time-dependent degradation pattern extraction, maximization of the information present within data arrays. In particular, the proposed method enables the generation of degradation maps that represent the distribution across the analyzed area of spectral changes induced by chemical modification due to aging. They enable a clear visualization of dye degradation within wool fibers.

Degradation maps were obtained starting from multimodal diffuse reflectance and fluorescence hyperspectral data from different samples, followed by chemometric-based multiblock data fusion and multivariate image segmentation. On these outcomes in mind, a pixel-by-pixel fitting model was developed and applied on fluorescence hyperspectral imaging data set to obtain degradation maps indicative of spatial variation of the dye throughout samples, exploiting emission intensity variation and spectral shifts and enhancing their role for the early detection of chemical modification of the dye. On the other hand, it is worth noting that the present research was not aimed to describe the specific degradation mechanism occurring to IC.

The approach led to the maximization of small spectral differences, enabling the characterization of minor alterations and their localization to specific fibers in the yarns analyzed, thanks to the high spatial resolution of the setup. The capabilities of this system have unveiled new possibilities in textile research and conservation. While the focus of this study has been on IC, the methods are applicable to a wide range of natural and synthetic dyes as well as various time-dependent processes where small modifications occur over time, inducing minor spectral changes that need to be detected.

Moreover, we demonstrated how this strategy enables more sustainable conservation of precious and fragile historical textiles. Indeed, the ability of noninvasively analyzing textiles and identifying the onset of a degradation process potentially enables the evaluation of the most suitable conservation/exhibition conditions. This will lead to reducing risks and the rate of aging and, consequently, minimizing the need for the artifact to undergo restoration. Future studies will focus on increasing the number of samples and varying the aging conditions, particularly with shorter irradiation times, in order to obtain relevant information on the initial spectral changes for the early detection of degradation.

## Supporting Information Available

Molecular structure of indigo carmine dye, detailed insights into the experimental setup for HSI using a Fourier transform-based TWINS interferometer, frequency distribution histogram of PC1 scores and PC1 loading profiles, average fluorescence spectrum of undyed wool sample before and after artificial aging, fitted average fluorescence spectra of undyed wool fibers and their fitted parameters, average fluorescence spectra of three clusters modeled as skew normal distributions, and the boundary conditions and starting values for the nonlinear fitting procedure applied to the F-block data (PDF)
