# Supplementary material for: Multimodal HSI Combined with Multiblock Data Fusion: A New Tool for the Study of Time-Dependent Alteration Processes in Dyed Textiles
Source: Anal Chem. 2024 Dec 6;96(50):19939–46. doi: 10.1021/acs.analchem.4c04236 (PMC11657530; doi:10.1021/acs.analchem.4c04236)
Supplement: Supplementary file 1 — ac4c04236_si_001.pdf [file ac4c04236_si_001.pdf]

Multimodal HSI combined with multiblock data fusion: a new tool for the study of time-dependent alteration processes in dyed textiles

Zelan Li[1], Alessia Candeo[2]*, Emilio Catelli[1], Marta Ghirardello[2], Paolo Oliveri[3], Cristian Manzoni[4], Silvia Prati[1], Daniela Comelli[2], Giorgia Sciutto[1]*

1: Department of Chemistry "Giacomo Ciamician", University of Bologna, Via Guaccimanni 42, 48121, Ravenna, Italy

2: Department of Physics, Politecnico di Milano, Piazza Leonardo da Vinci, 20133, Milan, Italy

3: Department of Pharmacy, University of Genoa, Viale Cembrano 4, I-16148, Genoa, Italy

4: IFN-CNR, Piazza Leonardo da Vinci 32, 20133 Milano, Italy

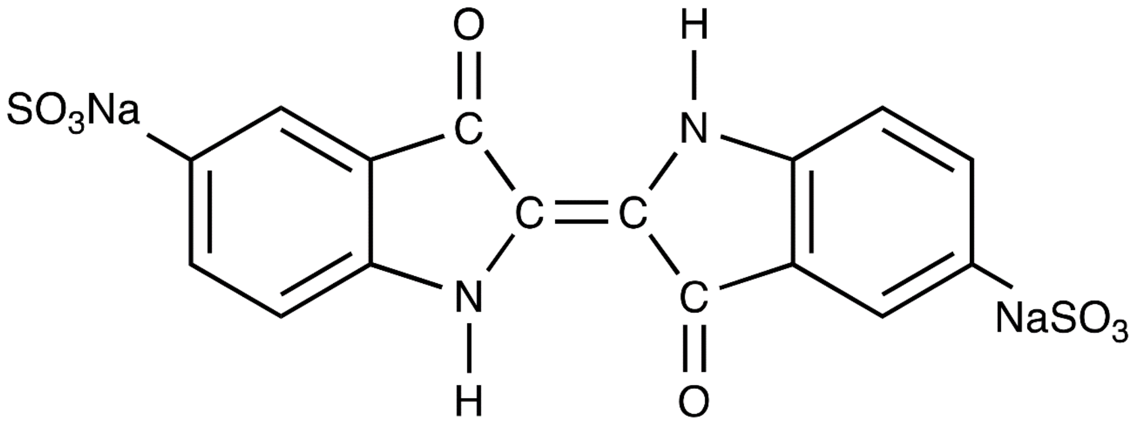

Figure S1: Molecular structure of indigo carmine.

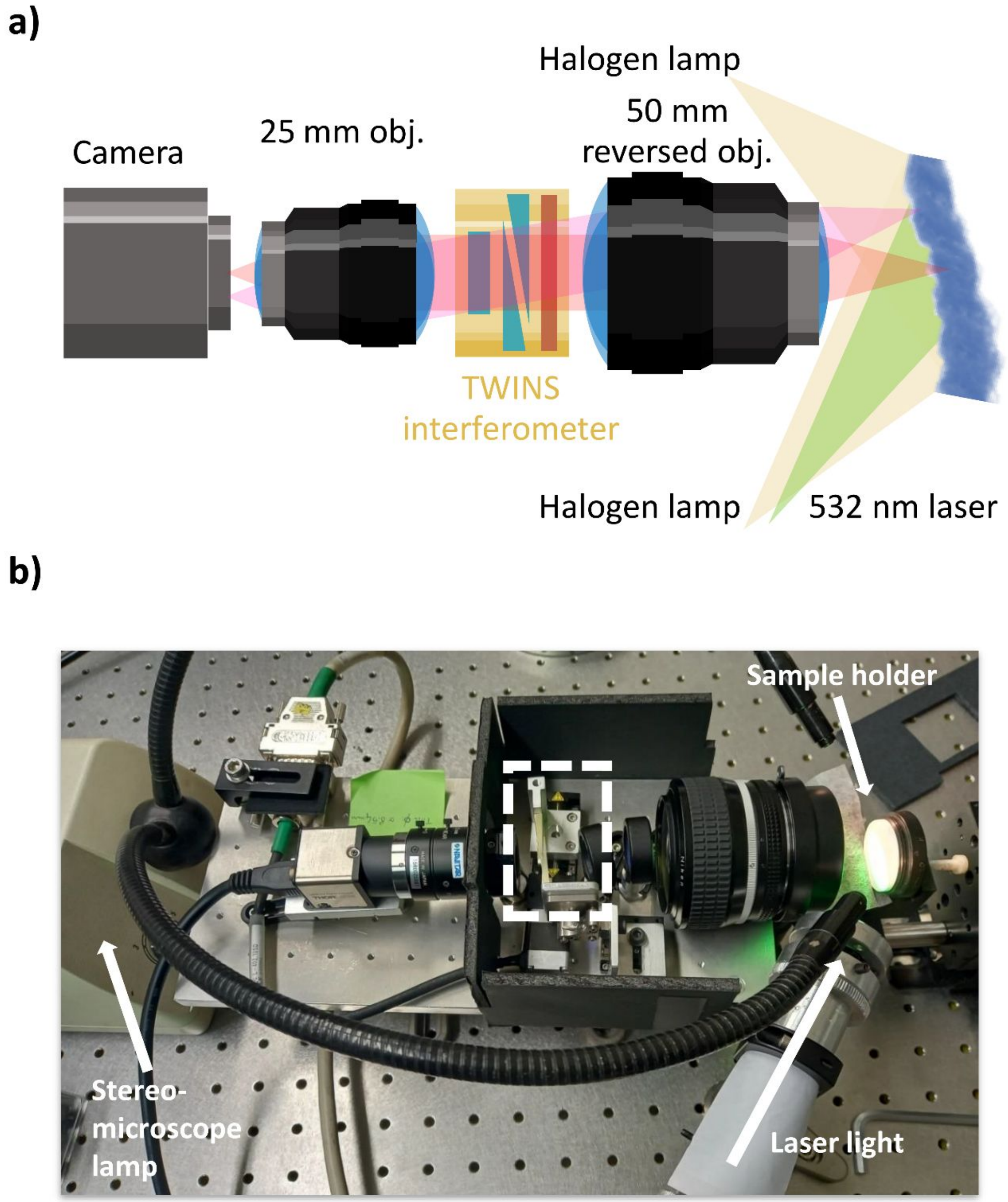

Figure S2: Experimental setup for HSI using the Fourier transform-based TWINS interferometer. a) Schematic diagram showing the HSI system configuration with a TWINS interferometer and a monochrome camera; b) photograph of the actual setup, showing the overall arrangement including the camera, interferometer, sample holder, and lighting sources for both diffuse reflectance and fluorescence excitation.

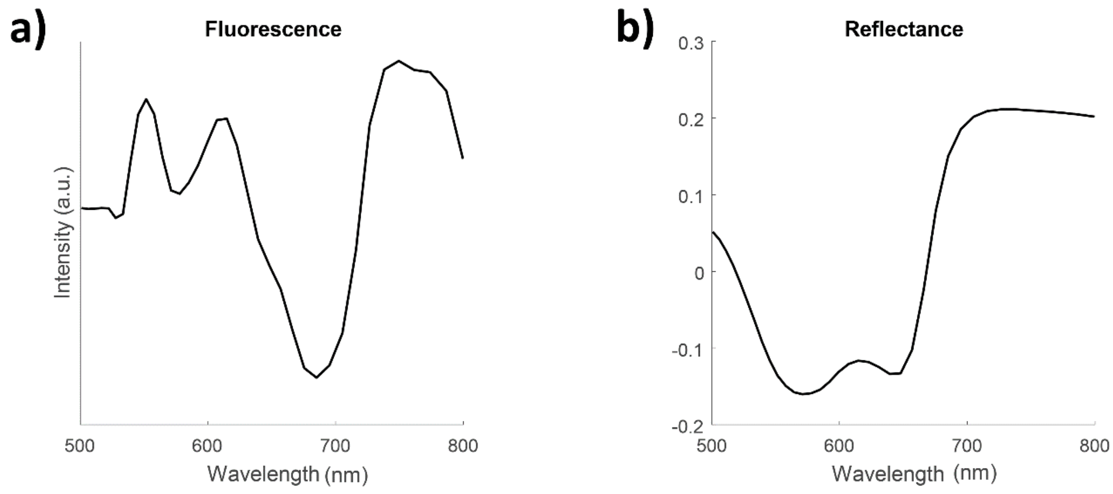

Figure S3: Loading profile extracted from PCA of the F&R block data performed after pre-processing.

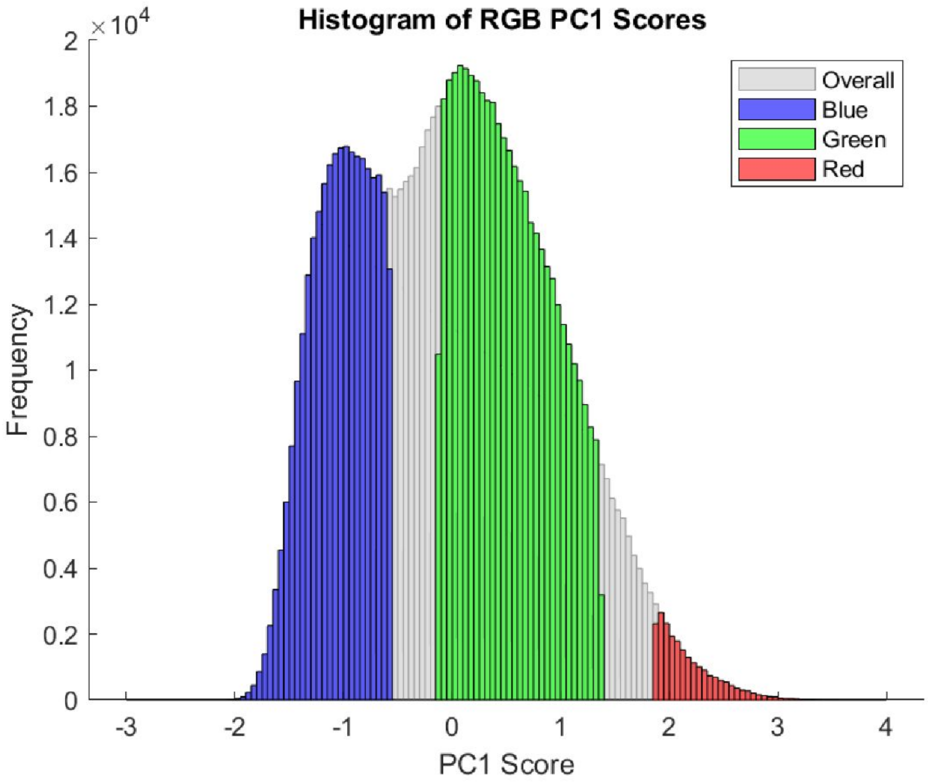

Figure S4: Histogram showing the frequency distribution of PC1 scores of the F&R block data. Colors represent threshold values set for the RGB clusters in data segmentation.

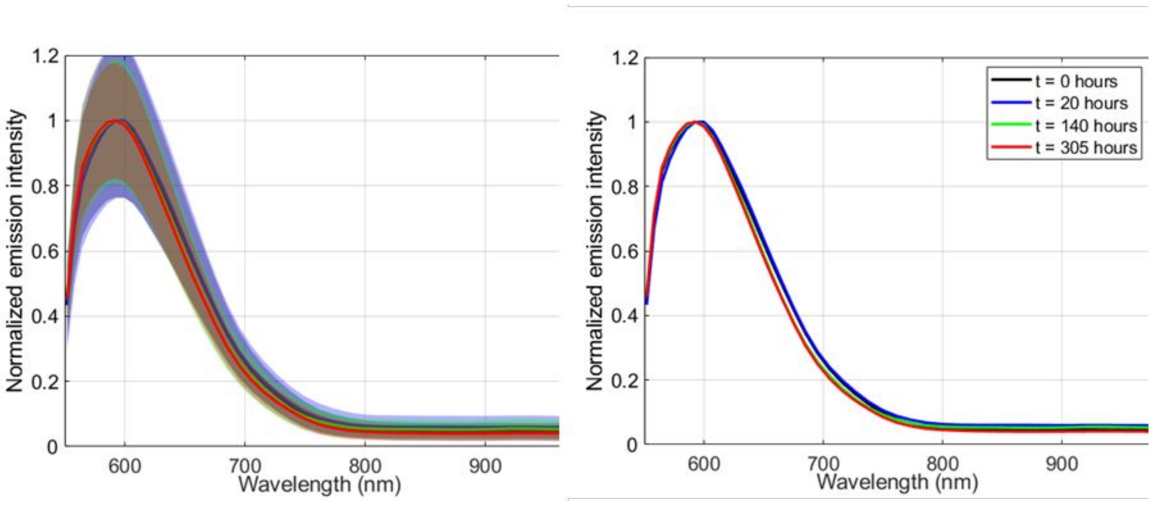

Figure S5: (Left panel) Average fluorescence spectrum of undyed wool sample (t = 0 hours) and undyed wool samples artificially aged for 20, 140 and 305 hours. Standard deviation within each sample is displayed as shaded area. (Right panel) To better appreciate changes among fluorescence spectrum of different undyed wool samples, average fluorescence spectra are plotted without standard deviation. It is noted that udyed wool samples display a narrowing of their emission spectrum of few nanometers following aging.

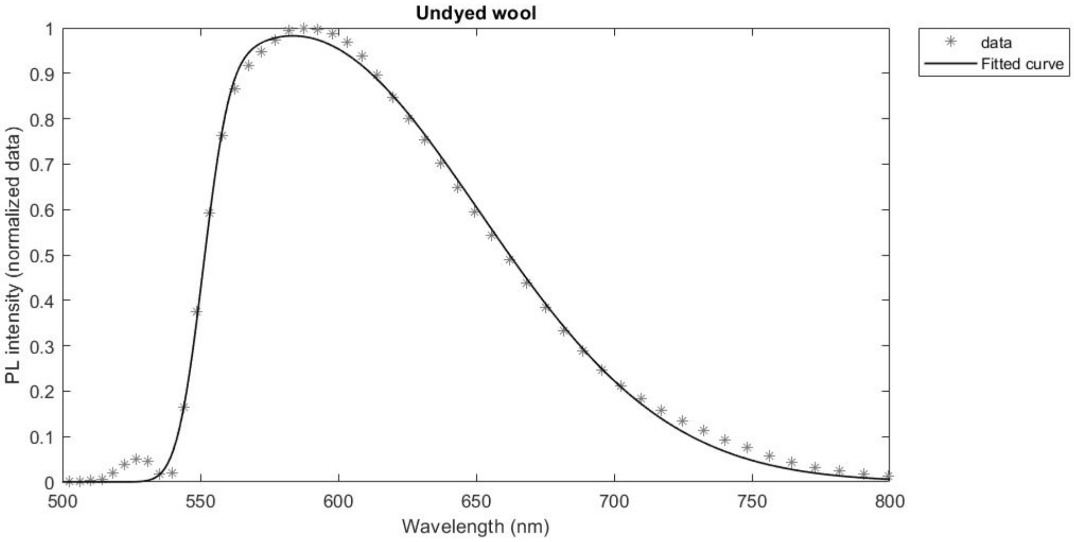

Figure S6: Average fluorescence spectrum of undyed wool fibres (grey asterisks) fitted with a skew normal distribution (continuous black line), with fitted parameter values provided in Table S1.

Table S1: Results of non-linear fitting of the average emission spectrum of undyed wool modelled as a skew normal distribution

	A_w [a.u.]	$\lambda_{MAX,w}$ [nm]	σ_w [nm]	p_w [nm]	s_w [nm]	R^2
Undyed wool	0.98	583.5	95.6	550.3	10.6	0.9977

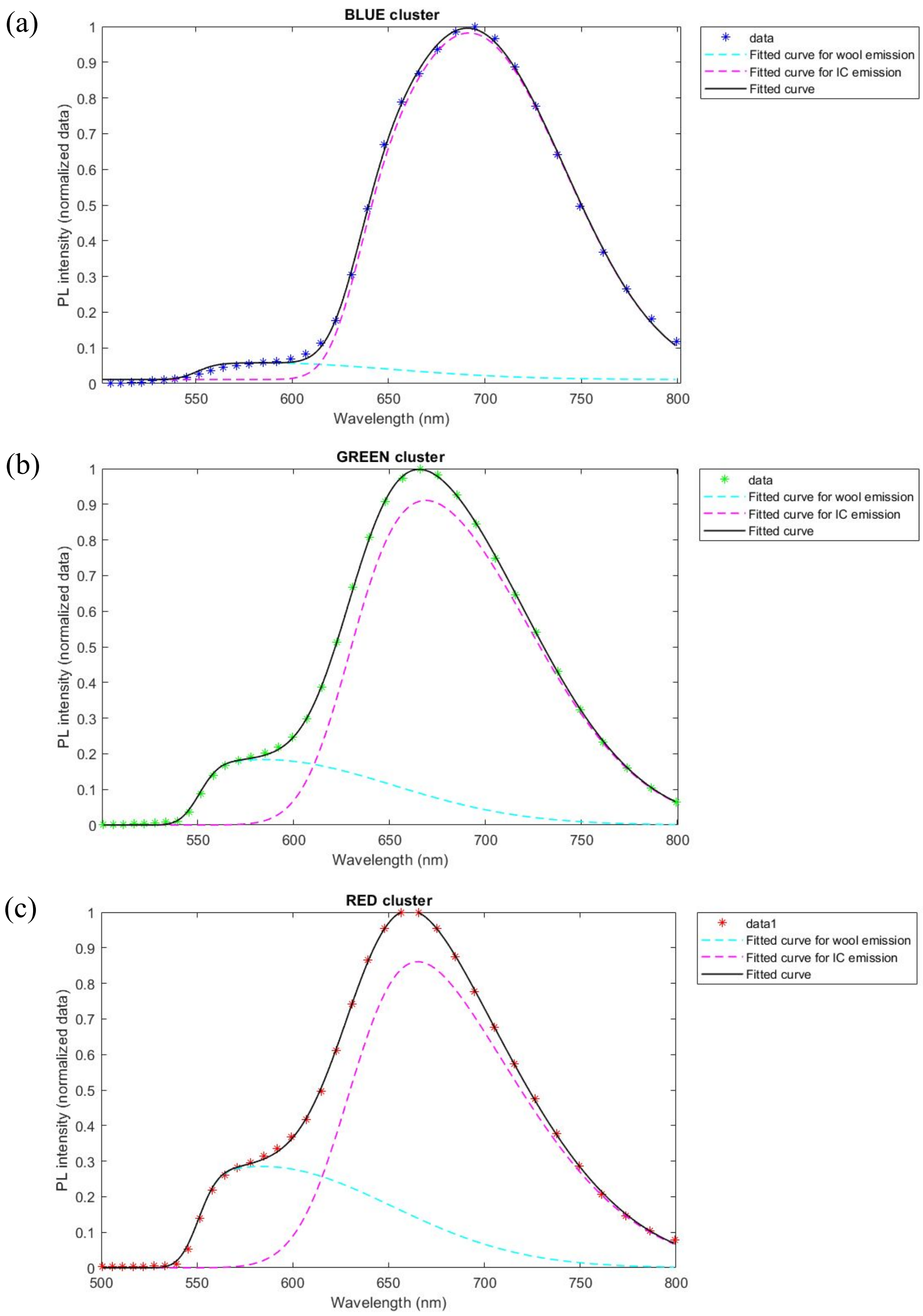

Figure S7: Average fluorescence spectrum of (a) blue, (b) green and (c) red cluster (blue, green and red asterisks, respectively). Each spectrum was modelled as the superposition of two skew normal distributions (continuous black line) accounting respectively for wool (cyan dashed line) and IC emission (purple dashed line). Fitted parameter values are provided in table S2.

Table S2: Results of non-linear fitting of the average emission spectrum of the blue, green and red cluster created following PCA-based segmentation of the whole F&R block dataset.

	$\lambda_{MAX,IC}$ [nm]	σ_{IC} [nm]	p_{IC} [nm]	s_{IC} [nm]	A_{IC}/A_w	R^2
Blue cluster	691.7	70.3	627.8	19.0	20.8	0.9994
Green cluster	662.7	83.4	623.2	29.2	5.1	0.9998
Red cluster	636.4	98.9	629.9	33.3	3.5	0.9997

Table S3: Boundary conditions and starting values of the fitting parameters of the non-linear fitting procedure applied to the F-block on a pixel-by-pixel basis

	**IC**					**wool**
	A_{IC} [a.u.]	$\lambda_{MAX,IC}$ [nm]	σ_{IC} [nm]	p_{IC} [nm]	s_{IC} [nm]	A_w [a.u.]
Low limit	0.5	630	60	620	15	0
Upper limit	1	710	110	635	35	0.5
Starting value	0.8	690	70	625	20	0.2
